# Organizational logics in time of crises: How physicians narrate the healthcare response to the Covid-19 pandemic in Swedish hospitals

**DOI:** 10.1186/s12913-022-08094-z

**Published:** 2022-06-03

**Authors:** Maritha Jacobsson, Maria Härgestam, Fredrik Bååthe, Emma Hagqvist

**Affiliations:** 1 Department of Sociology, Centre for Social Work, Uppsala University, Uppsala, Sweden; 2 Department of Nursing, Umeå University, Umeå, Sweden; 3 Institute for Studies of the Medical Profession, Oslo, Norway; 4 Institute of Stress Medicine, Gothenburg, Sweden; 5 Institute of Health and Care Sciences, Sahlgrenska Academy, Gothenburg University, Gothenburg, Sweden; 6 Unit of Occupational Medicine, Institute of Environmental Medicine, Karolinska Institutet, Stockholm, Sweden

**Keywords:** Neo-institutional theory, COVID-19, Discursive psychology, Healthcare, Management, Pandemic response

## Abstract

**Background:**

The COVID-19 pandemic has challenged healthcare organizations and puts focus on risk management in many ways. Both medical staff and leaders at various levels have been forced to find solutions to problems they had not previously encountered. This study aimed to explore how physicians in Sweden narrated the changes in organizational logic in response to the Covid-19 pandemic using neo-institutional theory and discursive psychology. In specific, we aimed to explore how physicians articulated their understanding of if and, in that case, how the organizational logic has changed during this crisis response.

**Methods:**

The empirical material stems from interviews with 29 physicians in Sweden in the summer and autumn of 2020. They were asked to reflect on the organizational response to the pandemic focusing on leadership, support, working conditions, and patient care.

**Results:**

The analysis revealed that the organizational logic in Swedish healthcare changed and that the physicians came in troubled positions as leaders. With management, workload, and risk repertoires, the physicians expressed that the organizational logic, to a large extent, was changed based on local contextual circumstances in the 21 self-governing regions. The organizational logic was being altered based upon how the two powerbases (physicians and managers) were interacting over time.

**Conclusions:**

Given that healthcare probably will deal with future unforeseen crises, it seems essential that healthcare leaders discuss what can be a sustainable organizational logic. There should be more explicit regulatory elements about who is responsible for what in similar situations. The normative elements have probably been stretched during the ongoing crisis, given that physicians have gained practical experience and that there is now also, at least some evidence-based knowledge about this particular pandemic. But the question is what knowledge they need in their education when it comes to dealing with new unknown risks.

## Introduction

During high burdens on healthcare services, organizational resources, support, and leadership are especially important to adapt to the crisis [1] and to reduce the risk of employees' ill health [2–5]. However, the COVID-19 pandemic has stressed and challenged resilience and perseverance in healthcare services. Research informing system adaptation and organization of resources and care during a crisis is urgently needed as many sectors were ill-equipped to meet the COVID-19 pandemic [6]. In the initial phase of the pandemic, healthcare workers needed to rapidly adapt and adjust to the COVID-19 landscape and adverse health effects were identified a few weeks into the pandemic [7–9]. Qualitative studies indicate that physicians experienced a continued lack of resources [8], uncertainty and challenges working in a new context [10], and legal and ethical dilemmas [8].

In a recent review, Sriharan et al. (2021) state that for crisis leadership, managers function at the intersection of the task, people, and adaptive competencies are essential and that "political, structural, and cultural contexts influence the demonstration of these competencies" ([1] (p. 9)). In this study organizational response to a crisis in a political, structural, and cultural context in Sweden are at focus. Sweden is divided into 21 self-governing regional authorities called Regions, responsible for providing a significant proportion of all public healthcare services in hospitals and primary care facilities [11]. The Regions are governed by political assemblies that have a considerable degree of autonomy. Every fourth year the political assemblies are elected in regional elections. The regional political assembly has the highest responsibility to provide medical care for their population and the power to decide how this should be done. All Regions also have an administrative and executive office. Thus, all 21 Regions could have different organizational structures to the healthcare services aligning to different political leadership [11]. To find common strategies between the Regions, a national structure organizes the 21 Regions called the Swedish Association of Local Authorities and Regions (SALAR). SALAR is an employers' organization that represents and advocates local government in Sweden. All of Sweden's municipalities and Regions are members. Regions are governed by the Healthcare Act for healthcare service delivery [12]. For national public health issues and diseases control, the Public Health Agency of Sweden draws guidelines that Regions can but are not obliged to follow.

To the best of our knowledge, most studies on the healthcare services during the COVID-19 pandemic have focused on the working conditions, workload, and health of healthcare workers, and less focus has been directed to how the physicians experienced organizational decisions and how the communication during prevailing circumstances was carried out. These aspects are essential to consider in strategies and policies to improve workforce planning, capacity, and safety in future crises [6]. As a response to this gap, this study aims to make use of neo-institutional theory [13–16] and discursive psychology [17–19] to explore how physicians in Sweden narrated the organizational response to the COVID-19 pandemic. In specific, we aim to explore how physicians articulated their understanding of *if* and, in that case, *how* the organizational logic has changed during this crisis response.

### Theoretical approach

**Fig. 1:**
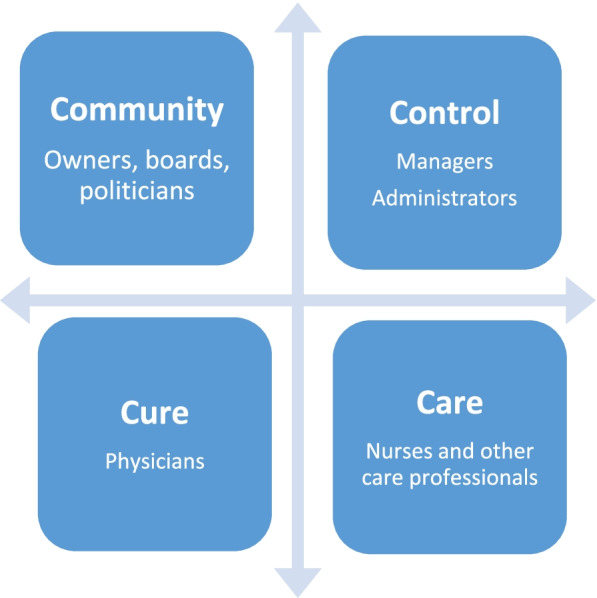
The four worlds in the healthcare organization, adapted from Glouberman and Mintzberg (2001) [20]

Within the neo-institutional theory, organizations are regarded as complex systems depending on, or influenced by, what is happening in the organizational environment and the broader (welfare) society as well as inside the organization [13–16]. There are many layers of decision-making on different levels in healthcare organizations. As mentioned above, there are the national governmental organizations, the Regional political assembly, the executive office, the organizational administration, and the level at which patients and physicians interact (see Fig. 1 below). Additionally, decisions are influenced by social and interpersonal relations inside and outside the workplace and population health. When a new phenomenon arises, such as the COVID-19 pandemic, social actors on different levels may have to make decisions that cannot be based on historical decision-making processes. To meet new demands brought by the new phenomenon, actors have to find new solutions.

Organizations can be described as physical entities and the ideas that permeate them [16]. Institutional logic is a concept within the neo-institutional theory that is useful in our study [21]. Thornton, Ocasio & Lounsbury (2012) define institutional logics as "socially constructed, historical patterns of cultural symbols and material practices, including assumptions, values, and beliefs, by which individuals and organizations provide meaning to their daily activity, organize time and space, and reproduce their lives and experiences" ([22] (p. 2)). Ocasio and Gai (2020) contend that the logic perspective can offer a way "to understand the factors that guide the beliefs and behaviors of actors" ([21] (p. 267)). Institutional logic determines what is considered to be appropriate or inappropriate behavior, to what values the employee adheres and defines what the focus of the organization is.

We define organizational logic as a part of the overall institutional logic (in an upcoming article, we are also going to study the medical logic, which we also define as a part of the overall institutional logic). Studying physicians' understanding gives an understanding of *if* and in that case, *how* they express their experiences on how the working conditions have changed during the response to the COVID-19 pandemic. As Thornton, Ocasio, and Lounsbury (2020) express, "social actors are in fact key to understanding institutional persistence and change" ([22] (Chapter 4, p. 1)).

Rules, routines, and values give legitimacy, stability, and meaning to how individuals act and communicate within the organizations. Scott (1995) talks about three elements that both structure and constrain behaviors: *regulatory*, *normative,* and *cognitive* [15]. *Regulatory* elements (how to do) are laws and regulations that set the framework for the activities within the organization. The *normative* elements (ought to do) are more prescriptive and are based on standards, values, and norms that will guide members within the organization. *Cognitive* elements (want to do) are about cultures and routines that are taken for granted, the "common sense". Normative and cognitive elements mean that individuals in organizations pursue a learned behavior. The regulatory elements should regulate and limit "incorrect" behaviors. When regulative elements are weak more informal structures are formed. These elements offer legitimacy by being legally sanctioned, morally authorized, and culturally supported.

Aligning with the neo-institutional theory that organizations are complex, Glouberman and Mintzberg (2001) provide a way to depict the complexity in healthcare. They suggest that hospitals are differentiated into four worlds: community, control, cure, and care, reflecting four different mindsets and four ways of thinking about organizing healthcare ([20] (Fig. 1)). These four unreconciled worlds can also be understood as professional identities. As long as the four worlds are disconnected, they argue, nothing fundamental will change.

Cure is the world of physicians, the medical community with clinical responsibility for medical decisions. It is the domain of superior expert knowledge based on licensed professional education and experience, even if treatment can be executed by other occupational groups [20]. Physicians are governed by regulative, normative, and cognitive elements, fostering a professional identity that includes a certain degree of autonomy in their work. To a large extent, they can act independently when it comes to medical decisions [23] and how to organize the work. In the world of care, nurses and other health professionals provide care to the patients and execute treatments delegated by physicians.

In the world of control, managers and administrative staff play a central role. They do not need a medical license but should follow regulative elements such as laws and policies like professionals in the community world. Managers have formal authority with overall and individual accountability and are expected to cope with changing demands from the community, such as access to healthcare, person-centred care, quality and security for the patients, and financial control. Glouberman and Mintzberg (2001) mean that management by numbers is considered essential and that many managers' approach to change is top-down, following linear and instrumental planning rationality [20]. In the community world, we find stakeholders, such as owners, public agencies, and political or interest-based lobby groups. Some of them are closely linked to the hospital, and others are more remote.

Glouberman and Mintzberg (2001) argued that the four worlds (Fig. 1) are divided by a horizontal and vertical cleavage ([20] (p. 59)). The horizontal is the great divide of healthcare, separating those who work clinically from those who do not. Below the horizontal cleavage, professional requirements and technological imperatives reign, and above are those "sensitive to the needs for fiscal control" and reform friendly. The vertical cleavage separates nurses and managers on one side, working with coordination and optimization for the hospital, from physicians who engage in individual patients and politicians who engage with a keen eye towards attracting future voters. The two most powerful worlds are managers and physicians with different power bases. Managers have a positional power in controlling the resources, and physicians have the power of exclusive medical expertise.

In conclusion, with inspiration from neo-institutional theory, we can talk about an organizational logic in healthcare that is quite multifaceted and that the communication between actors on different levels can be challenging. What happens when needing to respond to a crisis? Suppose there are changes in the organizational logic, is it the positional power from the community and control worlds, on a macro level, that influences the physicians' world on a micro-level? Is it the micro world, physicians' power of exclusive medical expertise, that is influencing the macro level?

The neo-institutional theory that Meyer and Rowan (1977) and DiMaggio and Powell (1983) developed attributed agency a limited role, but Thornton, Ocasio, and Lounsbury (2012) state that, studying social actors are central to understanding institutional persistence and change ([13, 22, 24] see also [25]). In order to study how actors such as physicians, in interviews construct their versions of *if*, and in that case *how* the organizational logic has changed during the pandemic, we are inspired by discursive psychology, which means that we are studying how the interviewed physicians construct their versions on how the organization changed during the initial phase of the pandemic.

Discursive psychology has its roots in social psychology and post-structuralism [26]. Language is not just treated as a transparent medium, instead, language constitutes knowledge and constructs versions and knowledge of how the so-called reality can be understood. The purpose of discursive psychology is to study how people construct their understanding of the world linguistically and how the speaker positions themselves in relation to others verbally. Theory and method are linked together. By using discursive psychology in combination with the neo-institutional theory, we can discuss and explore psychological phenomena in relation to organizational logic (for the combination of discursive psychology and organizational studies c.f.: [27–34]). A central concept in discursive psychology is "subject position" which is defined as the individual's "location within a conversation" [19]. This means that individual positions are adopted and become relevant within a specific conversation. Wetherell emphasizes the individual's multiple positions and the possibility of showing a variety of available subject positions negotiated in talk and interaction [17]. Parts of previous positions persist in the current situation and could be seen as sedimentation of past discursive practices [35]. The individual can vary positions within a conversation as well as between conversations, which means that language both produces and is produced of different discursive. Meaning that individuals position themselves or others in a preferable position (untroubled) or be positioned in a not preferable position (troubled) [17, 36]. In the talk, people are flexible when describing their versions of a phenomenon, and Wetherell uses "interpretative repertoires" to demonstrate how the individual has access to a variety of different repertoires to construct their version of reality [17].

## Method and data

### Aim

The aim of this study is to make use of neo-institutional theory [13–16] and discursive psychology [17–19] to explore how physicians in Sweden narrated the organizational response to the COVID-19 pandemic. In specific, we aim to explore how physicians articulated their understanding of *if* and in that case *how* the organizational logic has changed during this crisis response.

### Design

This study applies a qualitative research design inspired by neo-institutional theory and discursive psychology to gain in-depth knowledge of Swedish physicians' experiences of the organizational response to the COVID-19 pandemic. The study gained ethical approval from the Swedish Ethical Review Authority (2020–02,433). All participants gave their consent to participate both verbally and written.

### Data collection

Invitations to participate in the study were advertised on social media and in the journal for physicians in Sweden, "Läkartidningen". Those interested contacted the research team and were sent a longer invitation with a description of the project and information about consent. All those who initially contacted the team also consented to be interviewed. Most (24) interviews took place in virtual meeting rooms and five in a location chosen by the interviewed physician. Data were collected between June and November of 2020 by two of the authors (EH and FB). A semi-structured interview guide was designed using discussion themes, supportive questions, and probes. Discussion themes derived from previous research of psychosocial working conditions and included organization of work, support, physician well-being, and management and changes in healthcare systems. The interview guide was tested in pilot interviews, and minor changes were made before the rest of the interviews were conducted. Due to early reports from Italy and China that healthcare professionals working with patients infected with COVID-19 showed symptoms of post-traumatic stress disease (PTSD) each interview was proceeded by initial questions screening for PTSD. None of the participating physicians showed signs of PTSD, and interviews could proceed. Interviews took between 60 and 90 min and were audio-recorded and transcribed verbatim by an external person.

### Participants

In this study, a total of 29 hospital-based physicians were interviewed. These physicians worked in hospitals with different geographical locations in Sweden and under different Regions. They were specialist or under specialist training in internal medicine (including infectious diseases), neurology, orthopedics, pediatrics, and anesthesiology. Their experiences of working as a physician ranged from eight to 27 years. Seventeen of the interviewed physicians were women, fifteen were living with a partner and had children, two were living alone with shared custody of children and two were single with no children.

### Data analysis

Initially, the authors read through the interviews and identified two logics to be of importance:

organizational, and medical professional logic. The material is very rich, and, in this paper, we concentrate on organizational logic to understand which factors guided Swedish physicians in the response to the pandemic (the analysis of the medical logic will be presented in an upcoming article).

Authors MH and MJ led the analyzing process. Because of COVID restrictions and the physical distance, all four authors could not meet face-to-face but communicated regularly to discuss the analysis and results throughout the analyzing process. To study how the interviewed physicians verbally constructed their versions of what happened during the pandemic we initially analyzed three of the interviews more thoroughly with central concepts from discursive psychology and discussed between authors. Next, we identified interpretative repertoires within the organizational logic and how the physicians positioned themselves and others in the organization.

The research process was abductive, combining induction and deduction [37]. Initially, we read the transcriptions of the interviews close to the text theoretically inspired by discursive psychology and neo-institutional theory. The analysis has, after that, alternated between textual analyses and theoretical interpretations.

In line with the aim, exploring how physicians articulated their understanding of *if* and in that case, *how* the institutional logic has changed during the response process, we seek to identify: What repertoires were they using, how did they position themselves as in relation to other leaders and how did they perceive that they were positioned by others?

## Findings

An overall result is that hospital-based physicians in Sweden faced a difficult and complex situation during the response to the COVID-19 pandemic as well as throughout the first wave. Many of the informants expressed that they were confronted with problems in terms of how they, as professionals in the healthcare organizations would organize care and cure during this time. They were confronted with an unknown disease with symptoms presented among patients who did not follow traditional utterances, making them clueless. They had no treatment guidelines, disaster plans, and little crisis management to handle a global pandemic with an unknown virus. This situation resulted in reactions from colleagues that surprised the interviewed physicians. As one of the informants expressed:

So, this pandemic has shown sides of people I know that I had not anticipated, I really had not anticipated it. (IP 17)

A common feature in the interviews was that physicians talked about "management" referring to all levels of leadership above their closest manager, sometimes also including the first-line management. Some interviewees made no difference between the political assembly, the regional administrative leadership levels, and the hospital managers. Others were more organizational literate. To handle this empirical diversity in the results, we have used the reasoning from Glouberman and Mintzberg (2001), previously introduced and depicted in Fig. 1, who talk about a great horizontal divide separating those who work clinically from those who do not [20]. We refer to the many levels of administration involved in the pandemic crises by using the generic term "management".

### Organizational logic

The organizational logic expressed in the interviewees by the physicians can, with inspiration from discursive psychology, be described as a configuration of three interpretative repertoires (Table 1): The management repertoire, the repertoire of work environment, and the risk repertoire. In these repertoires, the physicians talked about factors that are related to regulative, normative, and cognitive elements that affected their decisions and behavior and how their positions as physicians changed during the pandemic.

**Table 1:** Overview of the findings

Organizational logic	*The management repertoire*
	*The repertoire of the work environment*
	*The risk repertoire*

#### The management repertoire

All interviewees agreed that the management at all levels faced major challenges during the response to the COVID-19 pandemic. When the first stories of the coronavirus from Italy and China reached the physicians in Sweden, they were concerned by these reports and did not know what to expect next. The common feeling among the physicians related to uncertainty and overall confusion with this unprecedented situation:

What is happening, what should we do? Should we do something now or should we wait? Should we wait for orders nationally or regionally before acting or should we do it ourselves? (IP1)

The physicians almost immediately felt they did not know what to do and expect next. They expected information from the management or The Swedish Public Health Agency and a plan to act upon, but the management was also uncertain. Interviewees experienced that the management did not provide the necessary information, and the physicians expressed that their questions were met by silence.

In the hospital's management, it was chaotic and confusing, and conflicts about who should do what. It was a big mess. And it was worrying, problematic in the beginning, stressful to feel that there was no support from above and no one had any real plan. (IP11)

As the pandemic continued, the physicians experienced growing fatigue and increased frustration towards the management. They felt that they all were put in troubled positions without control. In the excerpt above, the physician is using extreme case formulations [38], for instance, "chaotic", "confusing", "worrying", "stressful" to formulate a situation when there was "no support from above". The use of emotional terms illustrates the personal despair that they went through at the beginning of the pandemic when healthcare organizations needed to respond to the crisis. The physicians described a work situation with long work hours and a heavy workload. Many of them worked extra hours almost every day since the care of COVID patients was demanding, and then they had to work extra on-calls shifts.

[…] we worked many extra shifts, double the number of nights and weekend on-call shifts during that period. I did not work all weekends but eight out of eleven and when people were ill, we needed to take additional extra shifts […] Then I felt frustrated for the first time, I felt that the managers took for granted that we would work the extra shifts. They sent out a request to us all and expected us all to answer. It became a type of competition to come up with the best reason not to take the on-call shifts. Then I was the most relinquished, this is not fair, can I at least get a thank you for taking all these extra on-call shifts while working much already. (IP25)

In the excerpt above, the physician talks about how they made "a type of competition" to avoid extra shifts and troublesome work, which meant they tried to position themselves in relation to their colleagues. Although, all physicians talked about how they had to take responsibility to quickly change their work and adapt to the chaotic situation. Instead of being appreciated for their work and the more troubled positions they came in, the physicians experienced that the management did not pay attention to their commitment. One of the physicians describes a meeting with the top management of one Region at the beginning of March 2020.

We were dealing with an acute pandemic, and then [a top leader of the Region] raised that 'Have you thought anything about the ergonomics for those who work at home?' I just sat and thought, is he for real? The ergonomics for those who work at home when staff is tearing their lives out for the hospital and patients are dying. Yes, I hope no one has spent too much time thinking of ergonomics for those who work at home, I thought. (IP29)

The physician in the excerpt above experienced that the management did not prioritize staff that had more troubled and risky work tasks than others. When patients infected with COVID-19 began to arrive at the hospital's emergency departments, the organization of care and patient flow was unstructured and inadequately coordinated. Samples for COVID-infection were acquired from patients, but initially, it was unclear how the tests were to be carried out and to whom. The absence of action from leaders and sometimes also from the Public Health Agency was strongly criticized by the physicians in the interviews. New routines and work tasks had to be initiated to cater to emerging needs of the COVID patients, and sometimes nobody knew who was responsible. Physicians described that individual hospital personnel took the initiative to start testing. (IP29).

In some cases, the physicians experienced that they, from their troubled positions, together with their colleagues, had the power to make rapid changes in the organization, and that this became supported by the management. In these cases, they felt that their positions changed and became less troubled. In other cases, when physicians took their initiatives, it collided with the management’s ideas, and their positions became more troubled. Below there is one example of how one of the interviewees expressed problems that arose when the unit sent an e-mail to the management group suggesting that they should have larger rooms for group meetings or use digital meeting rooms.

We later heard that when our suggestion was presented in the managements group meeting, they responded that this [the suggestion to use a larger room for reports] showed that the employees have no trust in the first line manager and that the workgroup was difficult. […] The person writing the e-mail was told that this is not how communication to the leadership should be managed. Then we responded, how should we respond when we have no meetings. (IP20)

In the analysis of the empirical material, fear of the unknown and losing control seemed to be essential aspects that sometimes led to disagreements and conflicts. There were several examples of conflicts between the healthcare professionals within departments and between departments about what ought to be done. In more than one interview, the interviewed talked about conflicts between medical personnel at ICU and other clinics or units about which patients were eligible for ICU. Other informants described disagreement between physicians at different clinics related to where the patients with COVID-19 should be isolated.

Something that has worked less well is the operating clinics […] who thought that COVID infected surgical or orthopedic patients should be handled by someone else. They have not built emergency rooms for COVID infected patients nor any rooms for infected patients at the unit. (IP2)

The characteristic of the physicians' experiences of the pandemic care is that previous knowledge was not applicable, and previous work experiences did not guarantee and ability to predict the development of the disease. In response, media reports, social media, and colleagues became influential to know what they wanted to do. Discussion forums in social media were described as vital to gaining knowledge and control over the situation and agency. Both empirical and non-empirical knowledge and information were shared in social media groups. These groups were also described as supportive, and they became a place where physicians could "meet" with colleagues in a similar situation and share their experiences. Furthermore, to gain more knowledge and updated information about COVID-19, formal and informal channels with other physicians, both nationally and internationally, were established. Some described calling friends and colleagues in southern European countries to ask about their experiences. Contact with physicians from affected areas was a vital source of support that helped the physicians in their troubled positions. At the clinics, daily physical meetings and seminars took place to update the state of the pandemic and to contribute with support in difficult cases. The interviewed physicians also described how they called their colleagues at work or after work to discuss and to exchange experiences of how the work around the patients could be organized. In specialties with few consultants, they made themselves available for questions at all hours to support their colleagues.

The analysis of the management repertoire is that the pandemic challenged the existing organizational logic when it comes to how the work should be reorganized in relation to regulative (what they have to do), normative (what they ought to do), and cognitive (what they want to do) elements [15]. Earlier socially constructed patterns on how to manage the healthcare organization could not be followed. As we have shown, the physicians had to improvise and change their ways of working in different ways, some with support from the management above, some without. Those who felt support from the management could change their positions and find new and rapid solutions. Those who felt no or negative reinforcement from the management above came instead in extremely troubled positions and had difficulty handling the crisis. In the analysis, we found that the physicians talked about three ways through which healthcare was: top-down, bottom-up, and grassroots organization.

The first way in which healthcare was organized can be labeled as a top-down organization. Interviewed physicians experienced that the management above organized work without including the physicians. One physician describes that a special intensive care unit (ICU) or patients with COVID-infection was built and ready to receive the first patients when the management made a "U-turn" and canceled the unit. The physicians concluded that:

Two days before opening the COVID-ICU, they (the hospital management) stopped it. They claimed there was no need for a COVID-ICU. We had many patients that should have been suitable for that unit. It was very weird and poorly communicated from the hospital management to us at the clinic. (IP11)

In this case, the physicians expressed that they were put in a troubled position by the management, and it became unclear who should have responsibility for the reorganization process. They came into situations they had never experienced before, and the management made decisions that made the work even more difficult. In a second way, in a mandated bottom-up organization, a few physicians were given the responsibility and mandate to solve the rapid change of organization, i.e., staffing, and duties at the new pandemic departments. The mandate was clearly expressed and supported by the management. One physician describes:

The leadership of the region assigned experienced physicians from different specialties to work on the organization of pandemic care. […] They were given a clear mandate from the management that they could do what was needed to do and select by who and when things were to be done. (IP14)

In these cases, the management put the physicians in troubled positions since they gave them full responsibility for the reorganization process. Although, this position also made it possible for the physicians to rapidly take control and try to find the best solutions with support from above. In the third example, which can be described as a grassroots organization, physicians themselves took charge and lead the transition to pandemic care without any mandate from their management. The physicians began to prepare departments and the emergency unit with necessary personal protective equipment (PPE) to receive patients. They would base their decisions on second-hand sources such as social media and colleagues in other countries and organized pandemic care ad hoc according to the resources available. In some contexts, this led to efficient decisions and effective organization:

I was so impressed, and many with me, how quickly everything happened. Suddenly, we had an extra ICU in a gym in the basement. Suddenly, a new oxygen tank was being built because someone had figured out that there would be a shortage of oxygen. It was so incredibly cool to see how everything suddenly happened when normally everything [in the healthcare organization] would take two years to negotiate and some thinking and then a small SWOT and now it was just banging on, in a few weeks so much happened. Can't we have this all the time in healthcare? (IP14)

In the grassroots examples, the physicians themselves took control over the situation but without support from above which made their positions more troublesome since the management later came with directions that did not comply with the measures that already were taken.

Our analysis shows that the pandemic challenged the existing organizational logic regarding how the work should be reorganized and that the physicians were put in troubled positions by the management in different ways. In all three examples (top-down, bottom-up, grassroots) the physicians had to rely on their judgments and find new rapid solutions since there were no existing regulative, normative, and cognitive elements they could follow. Earlier socially constructed, common-sense patterns on how to manage the healthcare organization had to be changed.

#### The repertoire of the work environment

As we have shown above, there were problems in the response to the pandemic and the reorganization process in relation to how to manage the work in hospitals. All physicians experienced rapid changes in the work environment, implying that their positions in the organization changed. The physicians experienced that the work environment nor the occupational health and safety was a prioritized area, and the distrust towards the management grew.

The work environment is prioritized low, one [the management] is far, far behind in how they consider the workforce. It is the numbers and the Excel sheets that count, and very little about what one contributes to the organization and the development of the organization and the capacity around it. […] this creates an even worse work environment which we need to monitor because the employer does not consider it but rather just continues as usual. (IP3)

In the analyzing process, we identified three aspects that were important in relation to the repertoire of the work environment for the physicians: organizational changes, extreme workload, and shortage of personal protective equipment (PPE).

As we have shown in the management repertoire, the physicians were forced to (in different ways) reorganize their own (and others) work, which naturally affected the work environment. The pandemic entailed a major organizational change related to tasks and the physical premises to separate "dirty" from "clean", i.e., patients with symptoms of a COVID-19 infection from those with no symptoms. Departments were moved, and areas rebuilt, sometimes leading to solutions that did not work in practice and therefore acquired additional changes or the re-rebuilding of new areas. As "new" departments were built or new ways of organizing patient flow were created, staff from all categories and specialties were moved from their regular work units. They left their workplace from one day to another often with no or limited introduction. For some of them, their work at the "new" department included new tasks and unknown routines. Physicians with specialties in geriatrics or neurology described how they suddenly were responsible for patients with medical conditions that they had not been in contact with since their education (IP4). At most ICUs, the work situation was described as chaotic, they did not know what they ought to do, and they felt that their positions in relation to their colleagues became problematic. The majority of the staff at COVID-ICU had been reassigned from other departments and had never seen or worked with respirators or dialysis machines used at the intensive care. The physicians in the interviews describe that "there were no time and resources available for training" (IP28). The work situation created anxiety and the effect on care. In the citation below, one of the physicians describes the ICU situation when crucial collaborations occurred with nurses who have never met/cared for intensive care patients before (IP23).

Quality of care, of course, will be affected when they put a nurse with a surgical specialty that has never seen a respirator or dialysis machine on COVID-ICU with a severely sick COVID-patient. Of course, that creates an issue of quality of care. But we tried to organize the work to minimize the risks using more senior consultants. An experienced ICU nurse coordinated the work and how other ICU nurses could help. (IP23)

The workload was a critical aspect of the work environment repertoire. Some physicians talked about an extreme workload while others had less than normal to do. Both situations created frustrating situations. Physicians at intensive care or at the emergency care units involved in the intense and stressful pandemic care were under an extreme workload. In contrast, as personnel from the operating theatre were transferred to COVID-ICU and the lack of anesthetic medications following the prioritization for patients at the ICU, physicians from surgical specialties was not able to carry out planned surgeries. Physicians in the surgical specialties experienced competition among them to carry out the few surgical procedures conducted. They described a fear of losing surgical competence and of the increasing numbers of patients that needed planned surgery. The changes in the work environment meant that the physicians felt that they had to make decisions that were not the best for some patients, which meant that they felt that they came in troublesome positions in relation to the patients.

In temporary "dirty" departments, patients with confirmed or suspected COVID infection were situated no matter what condition they had. Junior or resident physicians were often responsible for the patients on the "dirty" side at the emergency department. Support or consultations with senior physicians were often in protective gear through the telephone. In situations where junior and resident physicians needed help from superior physicians, not all of these senior consultants had met COVID-patients earlier and knew less about how the infection behaved than the resident physicians did. In combination with the senior nurses, the senior physicians were also responsible for several departments and therefore overloaded with work and could not always support the junior physicians directly.

The junior physicians worked so hard. Those junior physicians in first triage were put on those long shifts, working all day and then night. They stood out there in the shed [a shed outside the hospital building for potential COVID infected patients]. (IP18)

As the number of patients increased, more administrative tasks around each patient emerged. Due to the work overload, the documentation process was perceived as more stressful than usual. Some of the physicians said they were afraid of missing important changes in the patient's treatment (IP2), and there were examples when patients were "missing". One example was a young man coming to the emergency department with a headache. The junior physician suspected cerebral hemorrhage but needed further advice from the senior colleague. The senior physician was busy and promised to return the call but forgot due to workload. The junior physician left the shift and the case to the colleague. The senior physician was alerted to the young man during the night, who was found unconscious and diagnosed later with an extensive cerebral hemorrhage. (IP25).

The third aspect that was expressed in the work environment repertoire was the shortage of personal protective equipment (PPE) which became critical in spring 2020 (see also [8]). The interviewed physicians expressed that the management did little to secure access to PPE. PPE availability was not dimensioned for a pandemic, so the lack of equipment became obvious. Instead, many of the interviewed described how hospital staff took the initiative to buy PPE themselves at their local pharmacy or to manufacture their visors based on descriptions that were found on social media. There are also descriptions of local contractors that offered to "restructure" their production line to start production of necessary PPE. Still, in some cases, the Regions did not allow that solution. In some Regions, the management chose to "downgrade" the classification of required face masks to a less effective model of face masks due to limited access (IP16). The physicians interpreted this as their employers "played" with their lives or that the management did not care if the staff became infected (IP17). The experience of being replaceable is exemplified by one physician who expresses the following quote with the extreme case formulation [38] "canon food".

[…] one [the management] see healthcare staff as canon food, if one gets sick, we put someone else in that position. (IP3)

Interesting to note is that the physician in this excerpt uses the war metaphor "canon food" which can illustrate how the person positions himself in relation to the management. The physicians were expected to be soldiers, and their health had to be sacrificed to defend the significant threat.

Long work hours created a situation of exhaustion that further added to the challenge of personal protection. The PPE obstructed the care work around patients and made the communication more difficult both with the patient and co-workers. The visors limited the view and caused bruises and pressure liaisons, and there were also accidents when co-workers injured their heads bumping into monitors (IP23). One physician expressed that they could not "*keep clean hands when one is so tired*" (IP3). As the limited PPE ran out, upset feelings and anxiety arose, and disagreements concerning the prioritization of PPE became frequent between personnel categories. In discussion with the union, questions arose of opportunities to refuse to work as long as the employer did not provide high-level protective equipment.

The analysis shows that the organizational logic was transformed in relation to the work environment and that the physicians felt that their positions become troublesome in relation to their colleagues, their health, and the patients' health. There were no regulative elements on how the physicians should work in this situation which created complicated relations between colleagues who did not know what they ought to do in the actual situation. The normative elements about work norms, ethical considerations, and duty responsibilities were no longer self-evident.

#### The risk repertoire

The repertoire of the work environment is partly intertwined with the risk repertoire. To analyze the risk repertoire more deeply, we have found two dimensions they talk about. The first dimension was about the risk that themselves, their families, and the patients could be infected. They also talked about patient safety in relation to when the patients did not get the care they needed about their medical problems. The other dimension was about risk and fear for the future.

The risk for the physicians of being infected became apparent and came close to reality when colleagues became seriously ill and were admitted to the ICU. In spring 2020, a nurse in Sweden died due to a COVID infection. This was reported by colleagues as a workplace accident and made headlines in the media. Furthermore, physicians also described situations where the safety and health representatives at workplaces were afraid of reprisal by pursuing cases further (IP3). For other informants, the COVID infection was followed by complications with long-term sick leave and rehabilitation. The interviewed questioned their work, and some were even considering quitting the work. The situation was perceived to be out of control.

We have always thought of Sweden as an incredibly good place to be in, but now this image is changed. For real, this bubble has exploded and there is nothing. I can't trust my colleagues, I can't trust that the healthcare will take care of me if I become ill, they have not taken care of me when I was sick, they have not wanted to take care of me now when I still have symptoms. This is the largest crisis of my life. (IP17)

In the excerpt above, the physician use the extreme case formulation [38] "the largest crises in my life" and the metaphor "this bubble has exploded" to underline how chaotic the situation. The physician also expresses a pessimistic view of Sweden as a country that can have implications for the future. A discussion arose between the union and leaders at the Regions whether being COVID infected at work can be regarded as an occupational injury. The management did not agree (IP3), which confirmed the impression among interviewed physicians that the work environment and occupational safety and health for the healthcare personnel were not crucial for the leaders in the Region.

As the shortage of PPE grew and became critical, the staff had to restrict and distribute the use of PPE to the situations when they were needed. The problem was that no one could predict when those situations would be.

In the beginning, it was completely bloody awful, nothing existed, really, in the beginning, we had nothing […] When we were entering a room with a patient with suspected COVID, the nurse could say that now you can't take any protection because we need to save as the patient is not infected for sure. I got completely crazy because how the hell can someone know if the patient can transmit corona, and was the thing, we couldn't say who was transmitting and not. It was crazy, and I know I was angry. They were saved, I felt this worry, and I thought that I did not sign up for this […] I have not signed up to go unprotected to a patient with a really dangerous disease. It was fear in the beginning, but that also passed, and later we got protection. (IP18)

The limited supply of PPE affected how the distribution of PPE took place to the different departments at the hospitals. In the excerpt above, the physician expresses emotions such as fear and anger, which shows an example of the emotional distress that the physicians went through in the troublesome positions they felt put into. Some clinics were communicated to be "safer" than others concerning risks of being transmitted with COVID from infected patients. Physicians from the psychiatric (IP20) and pediatric (IP17) departments described several scenarios where the management did not consider their safety and health because their patients were considered not to be a risk. For instance, physicians described that some departments were not prioritized for access to PPE in contact with patients and their relatives. In pediatrics (IP17), children were considered not to be contagious for adults, and no consideration was taken given that the children were always examined in the company of parents who could be infected. In the psychiatric emergency department, patients were not separated between clean and dirty. They were not tested, although many of the patients, due to their mental state, could not describe if they had any symptoms of COVID-19. This sharp division between departments became apparent when the personnel at the psychiatric department collaborated with personnel from other clinics around the same patient in the same room, and personnel from other departments were wearing PPE.

Pretty weird situations arose when we, for example at the ECT, stood there close to patients and other staff from the psychiatric department. We were holding the ECT paddles, and then you need to be close to the patients. Then the anesthesiologist entered the room with full protection gear because they were on the ventilator and were considered to be exposed to COVID. And next to them was us, unprotected. So, frustration grew. (IP20)

The physicians expressed that they came in troubled positions in relation to colleagues due to access to PPT in risk full situations, creating conflicts in professional relationships. Although many of the physicians described conflicting feelings towards experiencing a pandemic, they would not have liked to be without. They appreciated everything they learned and their effort. However, they also expressed a deep worry for the future. They described that they felt abandoned, powerless, and without energy concerning what to do in the future and to all patients that will need care when the pandemic is over.

And then one thinks of the care depth, and that we will start to produce, and by week 40 everyone should have had their four weeks [of vacation], and then it is supposed to be produced as normal at the surgical clinic. And we don't have any beds for patients nor enough staff […] It is darkness, and the workload will be worse than ever before unless we have a second wave. (IP2)

Another worry was that the management might interpret healthcare personnel's capacity during the pandemic to be a "new" standard, which they will refer to after the pandemic.

Now we will need to head off the employer. Normally we have this number of patients but now we have had the double amount, and [the employer think] that this seems to work, doesn't it? If we previously said that we, as physicians, can't be responsible for more than five patients at the ICU and now [during the pandemic] we had ten. So [the employer will think that] evidently you can have more than five patients, you can have ten, so now you can continue with ten patients. Do you [refereeing to the interviewer] get this shift in standard? (IP3)

For the profession used to work with evidence-based medicine and science, the COVID-infection was a challenge when they had to deal with risks and invent how to treat the infected patients. They talked about how new standards were established. This condition could not be predicted, which was experienced as frustrating and frightening.

## Discussion

This study explored how physicians in Sweden narrated the changes in organizational logic in response to the COVID-19 pandemic using neo-institutional theory and discursive psychology. The result shows that as the pandemic started and patients with COVID-19 infection came in increased numbers to the hospitals, there was no time for thoughtful planning on either level in the healthcare organizations. All of the interviewed physicians expressed that their work situation changed dramatically due to an overall lack of knowledge related to COVID-19. They did not receive any new recommendations from the community and control world ([20] (Fig. 1)). Our analysis of interpretative repertoires and how the physicians positioned themselves in relation to the reorganization process in the initial response to the pandemic, have enabled us to explore a variety of understandings. We found three different repertoires related to organizational logic changes: management, work environment, and risk repertories. According to the management repertoire, the physicians felt that they were put in troubled positions by the management since they did not get any directions and had to rapidly find new ad hoc solutions. In the work environment repertoire, the physicians expressed that they, to a large extent, also were put in troubled positions by their colleagues, for example, when it came to the distribution of tasks and workload. In the risk repertoire, they talked about how they positioned themselves as troubled when their values and beliefs on how to handle risk situations were challenged. When the physicians felt that they were put in troubled positions (by their management and colleagues) and when positioned themselves as troubled they came into, what we interpret as *extreme troubled positions.* In the interviews, they also used extreme case formulations [38] to clarify and underline the problems in these extremely troubled positions.

It turns out that the uncertainty in relation to regulatory, normative, and cognitive elements [15] led to organizational change. The healthcare organization had to change since no regulative element could guide the physicians. One example is that The Public Health Agency did not have any relevant guidelines on how to handle new pandemic crises, and the physicians did not get relevant information on how they should get access to and manage PPE. If they got some information, it changed from one day to another or between hours, and the trust in the management dropped. The management's lack of occupational health and safety thinking was perceived as the management jeopardizing both the personnel's and the patients' health. The physicians could not lean on the normative element of what they ought to do in their medical profession. In their professional education or practical experience, they had not got any workable tools to handle such a situation. The cognitive elements were also challenged when there was no longer any cultural understanding or common sense about handling the situation. It was hard for the physicians to know what they wanted to do personally. The three elements that earlier constrained and supported the healthcare organization had to change, and new ad hoc solutions influenced the organizational logic.

The physicians described a "vacuum" that arose. Policies, work rules, moral and ethical responsibilities, values, and beliefs about how the work should be performed that earlier were relevant and provided meaning to the physician's daily activity were no longer obvious, and ideological dilemmas [8, 39, 40] occurred on how to handle the situation. The physicians had to try to find new solutions, and with little or no response from the management the reorganization processes during the response to the pandemic, seemed to be quite "ad hoc". The physicians, to a large extent, changed the organizational logic in the cure world ([20] (Fig. 1)). The organizational logic in healthcare is quite multifaceted, and the communication between managers and physicians is challenging. In conclusion, and with inspiration from neo-institutional theory, we see how historical challenges became extrapolated when a global pandemic with an unknown virus presented itself. The result showed many local variations of what happened when needed to respond to the pandemic. In some Regions, the organizational logic was changed by physicians with help from the formal managerial positional powers, and by top-down decisions from the macro level. At the same time, there were Regions where the actual change was driven from the micro-level, mainly based on physicians' power and medical expertise. The results show how the managerial vacuum about how to respond to the pandemic, that existed in certain Regions, was filled with initiatives from physicians who had to handle the clinical patient needs. The results also show that there has been an emergent organizing principle, based on local contextual circumstances. The organizational logic was being altered based on how the two powerbases (physicians and managers) were interacting over time. This result is well-aligned with recent organizational sciences taking its origin in complexity sciences where the unpredictable nature of human organizations has been a definitional prerequisite, attracting healthcare practitioners and researchers [41–44].

Since the 21 self-governing Regions have a considerable degree of autonomy, and as individual physicians have multiple social identities which enable knowledge acquisition from different sources the organizational logic changed in different ways in the Regions. Given that healthcare must deal with the ongoing pandemic and that they have to prepare for future unforeseen crises, it seems important that healthcare leaders discuss what can be a sustainable organizational logic. According to this study, there should be clearer regulatory elements about who is responsible for what in similar situations. The normative elements have probably been stretched during the ongoing crisis, given that physicians have gained practical experience and that there is now also, at least, some evidence-based knowledge about this particular pandemic. But the question is what knowledge they need in their education when it comes to dealing with new unknown risks.

What happens to the healthcare organizations and in the 21 self-governing Regions in Sweden in the future will be interesting to study. Di Maggio and Powell (1983) mean that institutional change can take place in three different ways; coercive (top-down), normative (expectations of what is right and reasonable), and mimetic (organizations copy each other) [24]. Although, a relevant question is whether the COVID-pandemic and the re-organization process that has been part of the crisis management have changed the organizational logic or whether this is only a temporary change.

## Conclusion

The overall conclusion of this study is that the organizational logic in Swedish healthcare changed when hospitals had to respond to the COVID-19 pandemic. The three elements; regulatory, normative, and cognitive [15] that earlier constrained and supported the healthcare organization changed during the pandemic. The physicians were using three different repertoires when they talked about changes in the organizational logic; management, work environment, and risk repertories. The physicians felt that they sometimes were put in troubled positions by their management and/or colleagues and that they also positioned themselves as troubled. When they were positioned both by their management and their colleagues in troubled positions, they came into *extremely troubled positions.* How the organizational logic changed was based on local contextual circumstances. The organizational logic was being altered based on how the two powerbases (physicians and managers) were interacting over time. This study presents important knowledge on healthcare response to the crisis that can inform policymakers when preparing for future crises.

## Data Availability

The interview data analyzed during the current study are available from the corresponding author on reasonable request. Due the sensitive content in the material data is shared with caution.

## Abbreviations

ECT: Electroconvulsive therapy
IC: Intensive care
ICU: Intensive care unit
IP: Interviewed person
PTSD: Post-traumatic stress disorder
PPE: Personal protective equipment
